# Lack of Teratological Effects in Rats Exposed to 20 or 60 kHz Magnetic Fields

**DOI:** 10.1002/bdrb.20316

**Published:** 2011-10

**Authors:** Izumi Nishimura, Atsushi Oshima, Kazumoto Shibuya, Tadashi Negishi

**Affiliations:** 1 Environmental Science Research Laboratory, Central Research Institute of Electric Power IndustryChiba, Japan; 2 Contract Testing Department, Nippon Institute for Biological ScienceTokyo, Japan

**Keywords:** magnetic field, intermediate frequency, nonionizing radiation, teratogenicity, organogenesis, rat, risk assessment

## Abstract

**BACKGROUND:** A risk assessment of magnetic field (MF) exposure conducted by the World Health Organization indicated the need for biological studies on primary hazard identification and quantitative risk evaluation of intermediate frequency (300 Hz–100 kHz) MFs. Because induction heating cookers generate such MFs for cooking, reproductive and developmental effects are a concern due to the close proximity of the fields' source to a cook's abdomen. **METHODS:** Pregnant Crl:CD(SD) rats (25/group) were exposed to a 20 kHz, 0.2 mT(rms) or 60 kHz, 0.1 mT(rms) sinusoidal MF or sham-exposed for 22 hr/day during organogenesis, and their fetuses were examined for malformations on gestation day 20. All teratological evaluations were conducted in a blind fashion, and experiments were duplicated for each frequency to confirm consistency of experimental outcomes. **RESULTS:** No exposure-related changes were found in clinical signs, gross pathology, or number of implantation losses. The number of live fetuses and low-body-weight fetuses as well as the incidence of external, visceral, and skeletal malformations in the fetuses did not indicate significant differences between MF-exposed and sham-exposed groups. Although some fetuses showed isolated changes in sex ratio and skeletal variation and ossification, such changes were neither reproduced in duplicate experiments nor were they common to specific field frequencies. **CONCLUSIONS:** Exposure of rats to MFs during organogenesis did not show significant reproducible teratogenicity under experimental conditions. Present findings do not support the hypothesis that intermediate frequency MF exposure after implantation carries a significant risk for developing mammalian fetuses. *Birth Defects Res (Part B)* 92:469–477, 2011. © 2011 Wiley Periodicals, Inc.

## INTRODUCTION

Although intermediate frequency (IF: 300 Hz–10 MHz) electromagnetic fields are widely used in industrial machines and domestic electric appliances, the health risks which are attributed to the exposure to these fields have not yet been sufficiently evaluated. The World Health Organization (WHO) conducted risk assessment of magnetic field (MF) exposures at frequencies <100 kHz and published Environmental Health Criteria (EHC) 238 (WHO, 2007). EHC 238 identifies that such MFs are considered to have stimulating effects on excitable cells present in tissues, such as muscles and nerves, by magnetically inducing electric currents or electric fields in the body. These criteria provide the basis of the International Commission on Non-Ionizing Radiation Protection (ICNIRP) exposure guidelines (ICNIRP, 2010); on the other hand, the EHC indicated an insufficient number of studies on primary hazard identification and quantitative risk evaluation of the IF MFs from 300 Hz to 100 kHz (Litvak et al., 2002; WHO, 2007). Such risk assessment was based on studies of MFs predominantly at power frequencies (50 and 60 Hz).

Earlier investigations regarding the effects of IF MF included epidemiological studies on adverse pregnancy outcomes resulting from the use of a video display terminal (VDT), and animal reproductive and developmental toxicity studies that employed surrogate MFs simulating a VDT. A few case-control studies suggested an elevated risk of spontaneous abortion following the use of a VDT (Goldhaber et al., 1988; Lindbohm et al., 1992). However, a pooled analysis did not indicate significantly increased risks for miscarriage, birth of malformed children, or low-body-weight fetuses (Parazzini et al., 1993; WHO, 1998). Results from rodent studies have shown no VDT-MF-related reproductive or developmental effect (Huuskonen et al., 1998a; Robert, 1999; Brent, 1999a,b).

Recent sources of IF MFs include induction heating (IH) cookers, inductively coupled power transmission (IPT; wireless/contactless power transfer) for industrial material handling machines or home appliances, and a variety of wireless communication systems (ICNIRP, 2008). IH cooking hobs emit a vertical, space varying IF MF consisting of a fundamental frequency of 20 to 90 kHz and related harmonics, accompanied by power frequency and related harmonic MFs (Yamazaki et al., 2004; Fujita et al., 2007). IPT systems commonly employ 10 to 50 kHz MF with a sinusoidal waveform to transfer electric power efficiently through the large air gap between a pair of coils (Boys et al., 2000; Budhia et al., 2009). In occupational settings, high-intensity IF MF emissions from instruments such as magnetic resonance imaging machines, induction heaters, and welding machines have also been reported (Stuchly and Lecuyer, 1985; Floderus et al., 2002; Decat et al., 2006).

Dawson et al. (1998) exposed male and female rats to very intense and graduated 0.095 to 0.95 mT, 10 kHz, sinusoidal (IPT-like) MFs and reported no adverse reproductive effects with three different sets of experiments. The three sets involved the exposure of pre-mating males only, premating females only, and 22 whole gestation days. With this study as an exception, most previous rodent studies and including recent ones in Korea (Kim et al., 2004; Lee et al., 2009), have exclusively adopted sawtooth waveform (VDT-like) MF at 20 kHz only and with relatively weak intensities of typically around 10 to 20 µT (peak-to-peak [pp]). Since the revision of the guidelines in 2010 (ICNIRP, 2010), the magnitude 20 µT(pp) has fallen below the ICNIRP exposure guidelines for the public (27 µT(rms) and is equal to about 76.5 µT in (pp) terms). Considering the ubiquitous IF MF sources other than VDT, the higher exposure doses used for animal studies are essential to identify possible health hazards and to reveal the threshold dose in an animal model, which is needed for effective health risk assessment.

The domestic use of an IH oven or cooker is now very popular. It generates MFs at a fundamental frequency such as 20 or 60 kHz and the associated harmonic frequencies are used for cooking. These MFs are emitted from a hob and are converted to magnetically induced current in a pan that produces heat for cooking. Although a leak field should be very weak when the apparatus is properly used, the location of the MF source (the hob) is close to the cook's abdomen. Therefore, reproductive and developmental effect is a concern.

We have demonstrated that up to 1.1 mT(rms), 20 kHz MF was not associated with teratogenicity in a chick embryo model (Nishimura et al., 2009). In this rodent study, we adopted IF MFs at 20 kHz in 0.2 mT(rms) and at 60 kHz in 0.1 mT(rms). These MF intensities reflect the maximum strength at our rodent exposure facilities for the respective frequencies. Because no precedent studies have repeatedly demonstrated the teratological effects of IF MFs in rodents, we chose feasible maximum intensities for hazard identification, with only two doses per frequency, but duplicated experiments to ensure reproducibility. The strength of 0.2 mT(rms) is 7.4 times greater than that of the current ICNIRP exposure guidelines for the public. The MF intensity experienced by a cook depends on the power setting of the IH cocker, pan size, and position of the cook, and intensity rapidly decreases in line with distance from the hob.

Although the exact frequency of fundamental IH MF varies across product models from different manufacturers, 20 and 60 kHz were adopted in this study as commonly employed frequencies of IH cookers on the market. The 60 kHz MF is also the third harmonic of the 20 kHz MF and has never been examined for reproductive and developmental toxicity with rodents. In addition, we used a sinusoidal waveform, because it is simple for other researchers to reproduce. A sawtooth waveform on the other hand has rather complicated field characteristics such as rise/decay times and polarities. Data reproducibility is one of the important issues in electromagnetic field research.

The aim of this study was to determine whether 20 and 60 kHz IF MF exposures have embryotoxic, fetotoxic, and/or teratogenic potential in rats. Referring to the OECD (2001) and ICH (1993) guidelines for developmental toxicity study, we exposed animals specifically during organogenesis unlike in earlier studies. The exposure experiment was repeated twice for each frequency to confirm the reproducibility of its outcomes. All examinations after necropsy were conducted by an independent, GLP-licensed laboratory in a blind fashion to ensure quality assurance.

## MATERIALS AND METHODS

### Animal Husbandry and Mating

The Institutional Animal Experiment Committee of the Environmental Science Research Laboratory, Central Research Institute of Electric Power Industry, approved all animal experimental procedures of this study in accordance with the Committee's guidelines under Japanese Law Concerning the Protection and Management of Animals.

This study comprised four independent but identical experiments (two with 20 kHz MF exposure and two with 60 kHz MF exposure) performed on different dates and conducted with different animal batches. The same protocol was used in all experiments.

Male and female, SPF/VAF, Crl:CD(SD) rats, 67 each, were purchased from Charles River Laboratories Japan Inc (Kanagawa, Japan) at the age of 11 and 9 weeks, respectively. The rats were quarantined for a week in a barrier system of an experimental animal facility where background MFs were extremely low (<0.001 µT(rms) at 20 and 60 kHz, and 0.03 µT(rms) at 50 Hz). Room air was HEPA-filtered, and room temperature and humidity were maintained at 23 ± 2°C and 50 ± 20%, respectively. The light cycle was 12 hr/12 hr (07:00–19:00 on). The rats were housed in polycarbonate cages (23 cm W × 33 cm D × 17 cm H; Tokiwa Kagaku Kikai Co Ltd., Tokyo, Japan) and fed with pellets (NMF, 15 kGy irradiated, Oriental Bioservice, Kyoto, Japan) and water (distilled, autoclaved, 10 ppm chlorine, adjusted to pH3 by 1N HCl) ad libitum. Commercial wood chips (White flak, 30 kGy irradiated, Charles River Laboratories Japan Inc) were used.

After the quarantine period, rats were weighed and mated (1 male vs. 1 female) for up to 4 days. Vaginal smears were checked every morning to identify copulated females and establish gestation day 0. Paired males were removed upon pregnancy confirmation. Pregnant females were individually bred and randomly assigned to either the MF-exposed (*n* = 25) or sham-exposed (*n* = 25) group by body weight at gestation day 0. The dams were moved to dedicated MF exposure facilities on gestation day 7.

### MF Exposure Facilities

A pair of exposure facilities (Takenaka Co, Osaka, Japan) for IF MF exposure (one for 20 kHz and another for 60 kHz) were built in an area where the background 50 Hz MF was extremely low at <0.02 µT(rms). Both facilities were identical with respect to appearance, layout, structure, and other room conditions. When one facility was used for an MF-exposed group, the other was used for the sham-exposed group without electric current in its exposure coils. The facilities were located 37.4 m apart to minimize stray IF MFs during exposures that were less than 0.001 µT(rms) at both 20 or 60 kHz as described later.

A sinusoidal 20 or 60 kHz signal was generated by a multifunction synthesizer (DF1906, NF Co., Kanagawa, Japan). The 20 kHz signal was amplified by a single power amplifier (4510, NF Co.), whereas the 60 kHz signal required two power amplifiers (BP4610, NF Co.). These signals were applied to the respective MF exposure coils situated in each animal exposure room. The electric currents required to produce maximum flux density were 7.0 to 7.2 A for 0.200 mT(rms) at 20 kHz or 13.4 to 13.8 A for 0.100 mT(rms) at 60 kHz. These currents were constantly maintained within a range of the target values ± 2%. Linearity between the input currents and the MF intensities was 1.000 in a range of 0 to 0.200 mT(rms) at 20 kHz or 0 to 0.100 mT(rms) at 60 kHz.

**Fig. 1:**
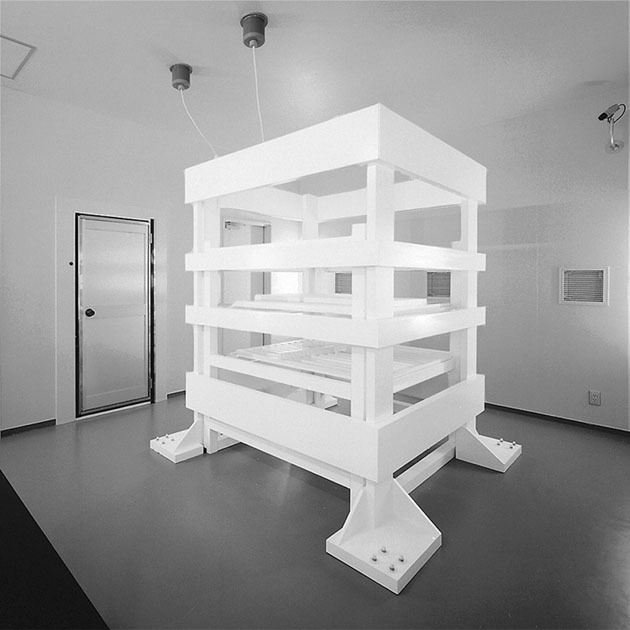
Intermediate frequency (20 or 60 kHz) magnetic field exposure system for small laboratory animals. Two exposure systems were built; one for 20 kHz and another for 60 kHz. Their appearances and dimensions were identical so that the systems were interchangeable for the sham-exposure (control) system. Coil wires for 20 or 60 kHz MF were installed in four horizontal wooden bobbins of the respective exposure system. Distances between the four coils were 60, 41, and 60 cm. The sides of the square coils were 150 cm, and the height of the top coil bobbin from the floor was 222 cm. The Merritt-type four-square coils could produce uniform (± 3%) 20 kHz MF up to 0.200 mT(rms) or 60 kHz MF up to 0.100 mT(rms) magnetic fields in a cubic space (100 × 100 × 100 cm) at the center of the coils where three wooden racks were layered to accommodate up to 36 rat cages in total. The magnetic field was vertical in orientation and sinusoidal in waveform.

A Merritt-type (Merritt et al., 1983), four-square coil system was adopted for both frequencies (Fig. 1). The four coils were layered horizontally to produce extremely uniform, vertical MFs in a large cubic space (100 × 100 × 100 cm) located within the coils. In the designated exposure space, three wooden racks were equipped to accommodate up to 36 rat cages with 12 on each rack in a 3-row by 4-column layout. One side of the square coils was 150 cm and the height of the four coils was 161 cm. The four coils were spaced at intervals of 60, 41, and 60 cm. Turns of coil wires were 26:11:11:26 for the 20 kHz MF exposure system and 7:3:3:7 for the 60 kHz one. These configurations were determined based on maximum MF uniformity. The MF variability within the exposure spaces was less than 3% in both coil systems. Although the coil wires for both 20 and 60 kHz MFs were different in material, thickness, and number of turns, they were installed in the same type of wooden bobbin boxes to ensure the appearance and dimensions of both exposure systems were identical.

The structural outer walls, high roof, and deep basement contained steel-bar reinforcing grids to reduce leakage of MF to outside of the facility without distorting the emitted MFs for animal exposure (Yamazaki et al., 2007). When the exposure coils were activated to produce the facility maximum MFs, the animals in the corresponding sham-exposed facility received less than 0.001 µT(rms) MF at 20 or 60 kHz and 0.03 µT(rms) at 50 Hz. These measurements were taken by commercial instruments: EFA-300 and ELT-400 (Narda Safety Test Solutions, Pfullingen, Germany) for IFs, and MFM-12A (Shoden Co., Tokyo, Japan) for power frequency. Geomagnetic field at the location was about 48.3 µT.

A wide-frequency-range (from 25 Hz to 100 kHz) MF meter developed by Yamazaki et al. (2004) and a digital oscilloscopes (TDS 3014B, Tektronix, Inc., Tokyo, Japan) were used to measure the harmonic components of the 20 kHz MF. ELT-400 and E4408B (Agilent Technologies, Santa Clara, CA) were used for 60 kHz MF. Harmonic components were negligible; 1/100 to 1/1,000 of each primary frequency.

### Exposure Procedures

Dams were individually housed in a cage and evenly distributed on the three racks. Cage position within and among the racks was changed twice a week to homogenize the possible effects of subtle environmental differences such as illumination and air flow. Animal husbandry in the MF exposure facilities was the same as that in the experimental animal facility except for the following factors: cage lids were made of polycarbonate, and glass water nozzles and pellet containers were used instead of metal ones to avoid induced current as a result of MF exposure.

Dams were exposed to 20 kHz, 0.20 mT(rms) or 60 kHz, 0.10 mT(rms) MF, or sham-exposed every day from gestation day 7 to day 17 for 22 hr/day (11:00 to 09:00). Electric current in the exposure coils was continuously monitored and recorded every 10 min with a data logger (IDSmart, Yamatake Co., Tokyo, Japan). Animal care including weighing and clinical observations were conducted daily during the unexposed 2 hr. Dams were returned to the experimental animal facility after termination of MF exposure and bred there until necropsy at gestation day 20.

### Teratological Evaluation

Dams and mated males were randomized, respectively, for sampling order, and the order was adopted for subsequent necropsies, hematological measurements, and pathological examinations. All teratological evaluations and clinical pathology were conducted blindly by Nippon Institute for Biological Science (Tokyo, Japan). Persons performing the evaluations had no knowledge of the animals' exposure status.

Dams were euthanized on gestation day 20. The abdomen cavity was opened and blood was drawn from the abdominal aorta. Abdominal and thoracic organs were examined. The uterus and attached ovaries were excised, and number of corpora lutea in each ovary was recorded. The uterus was opened and inspected for abnormalities. The fetuses and placentas were removed, blotted, and weighed. The number of live fetuses, dead fetuses, and implantation sites was recorded. Uteri were examined under an illuminated magnifier to establish whether complete or early resorption had occurred. Postimplantation losses during early, middle, and late pregnancy were, respectively, classified as the following: implantation sites on the endometrium or placental remnants smaller than 3 mm in diameter, placental remnants larger than 3 mm in diameter or dead fetuses smaller than the placental diameter, and macerated fetuses or dead fetuses larger than the placental diameter.

Live fetuses were identified by numbering them in sequence from the ovary end to the vagina end of the left uterine horn to the right regardless of sex. Fetuses with body weight <2.5 g were categorized as low-body-weight fetuses. Each fetus was examined for gross external abnormalities under an illuminated magnifier. Fetus sex was determined by measuring anogenital distance. Even-numbered fetuses and removed ovaries were placed in Bouin's fixative and examined for internal abnormalities. Odd-numbered fetuses were eviscerated then fixed in alcohol, and their skeletons were stained with Alizarin red S. The skeletons were then examined for abnormalities in size, shape, and ossification.

Mated males were euthanized and necropsied. Gross observations were performed and abnormalities were recorded. The testes, epididymides, prostate gland, and seminal vesicles were removed and fixed in 10% buffered formalin.

### Clinical Pathology

Collected blood was placed in tubes containing anticoagulant EDTA-2K. An automated hematology analyzer (SF-3000, Sysmex Co., Hyogo, Japan) was used to measure hematology determinations. The variables included hematocrit percentage, hemoglobin concentration, erythrocyte count, mean corpuscular volume, mean corpuscular hemoglobin, mean corpuscular hemoglobin concentration, platelet count, and leukocyte count. Differential leukocyte counts and the morphological evaluation of blood cells were microscopically performed on blood smears stained with Wright-Giemsa.

Blood for clinical chemistry evaluation was placed in tubes containing heparin sodium and centrifuged, and the serum was separated. Clinical chemistry variables were measured using an automated chemistry analyzer (7060, Hitachi Ltd., Tokyo, Japan). Endpoints included blood glucose, total protein, albumin, total cholesterol, phospholipids, neutral fat, total bilirubin, creatinine, blood urea nitrogen, aspartate aminotransferase, alanine aminotransferase, lactate dehydrogenase, alkaline phosphatase, creatine kinase, γ-glutamyltransferase, calcium, and inorganic phosphoric acid. Serum sodium, potassium, and chloride were measured with an electrolyte analyzer (EA07, Atwill Corp, Kanagawa, Japan).

### Statistical Analyses

Welch's *t*-test was used for statistical analysis of data between the MF-exposed and concurrent sham-exposed groups, with a level of acceptance of probability of *p*<0.05. Data included body weight of pregnant female, number of corpora lutea, body weight of fetus, placental weight, endpoints of hematology and blood biochemistry, and ossification. The Wilcoxon two-tailed test was used to compare preimplantation losses per dam, postimplantation losses per litter, total implantation losses per litter, and male to fetus ratio per litter between the groups.

Fisher's exact test was then applied to evaluate group differences for gross pathological findings of the dams. External, visceral, and skeletal findings (except for degree of ossification of fetus and incidence of low-body-weight fetus) were also analyzed with Fisher's exact test on a per dam or per litter basis. Differences between groups were considered significant at *p*<0.05, one-tailed test.

These statistical analyses were performed using SAS (SAS Institute Inc, Cary, NC) and EXSUS (Arm Co, Ltd., Osaka, Japan) programs.

## RESULTS

### Maternal Toxicity

No deaths occurred in any of the experiments during the study. Clinical observations did not reveal any significant findings. No gross lesions were noted at necropsy in any dams in any experimental group. No significant differences in dam body weight or body weight gain during gestational and exposure periods (gestation days 7–17) were found in any of the MF-exposed groups when compared to that in the concurrent sham-exposed groups (Table 1).

**Table 1:** Body Weight of Pregnant Rats Exposed to 20 or 60 kHz Magnetic Fields During Organogenesis

		20 kHz, 0.2 mT(rms) Magnetic field				60 kHz, 0.1 mT(rms) Magnetic field			
			
		Experiment 1		Experiment 2		Experiment 1		Experiment 2	
					
		Sham-exposed	MF-exposed	Sham-exposed	MF-exposed	Sham-exposed	MF-exposed	Sham-exposed	MF-exposed
Examined dams		25	25	25	25	25	25	25	25
Day of gestation	0	236 ± 13^a^	235 ± 13	234 ± 13	234 ± 13	243 ± 11	243 ± 10	257 ± 11	256 ± 11
	7	279 ± 13	277 ± 15	275 ± 15	274 ± 15	283 ± 13	282 ± 14	300 ± 14	299 ± 13
	18	365 ± 18	359 ± 20	356 ± 21	355 ± 21	368 ± 18	369 ± 16	387 ± 18	386 ± 18
	20	398 ± 19	391 ± 22	387 ± 23	386 ± 22	399 ± 21	398 ± 21	422 ± 20	416 ± 21
Weight gain									
Pregnancy period: days 0–20		162 ± 14	156 ± 13	154 ± 16	153 ± 11	156 ± 16	155 ± 17	166 ± 15	160 ± 21
Exposure period: days 7–17		86 ± 8	82 ± 11	81 ± 10	81 ± 11	85 ± 9	87 ± 9	88 ± 11	87 ± 12

MF, magnetic field.

a Mean ± SD (g).

Hematology and blood chemistry evaluations of the dams showed, with some exceptions, no significant group differences between respective sham- and MF-exposed groups across all experiments. Exceptions comprised a decrease in white blood cells in experiment 2 of the 20 kHz MF exposure, an increased percentage of neutrophil band formed in experiment 2 of the 60 kHz MF exposure, a decreased percentage of lymphocytes in the same experiment, a decrease in amounts of total protein in experiment 1 of the 60 kHz MF exposure, and a decrease in total bilirubin in the same experiment.

### Reproductive Performance and Prenatal Mortality

Reproductive performance of dams and prenatal mortality in the litters are given in Table 2. The mean number of corpora lutea per dam showed a significant difference between the sham-exposed and MF-exposed groups in experiment 1 of the 20 kHz MF exposure; however, the formation of corpora lutea preceded MF exposure. Further, the mean number of implants per dam in the experiment did not significantly differ between the groups, indicating that the observed difference in the number of corpora lutea did not affect teratological evaluation of the experiment. Other than the experiment, the number of corpora lutea per dam, number of implants per dam, and percentage preimplantation losses per dam did not significantly differ between the MF-exposed and the respective sham-exposed groups.

**Table 2:** Reproductive Performance, Prenatal Mortality, and Fetal and Placental Sizes in Rats Exposed to 20 or 60 kHz Magnetic Fields

	20 kHz, 0.2 mT(rms) Magnetic field				60 kHz, 0.1 mT(rms) Magnetic field			
		
	Experiment 1		Experiment 2		Experiment 1		Experiment 2	
				
Parameter	Sham-exposed	MF-exposed	Sham-exposed	MF-exposed	Sham-exposed	MF-exposed	Sham-exposed	MF-exposed
Examined dams	25	25	25	25	25	25	25	25
Corpora lutea per dam	16.6 ± 1.8^a^	15.4 ± 2.1*	15.3 ± 2.2	15.2 ± 2.3	16.2 ± 1.9	16.1 ± 2.5	16.2 ± 2.7	17.3 ± 2.5
Implants per dam	15.1 ± 1.2	14.4 ± 1.6	14.4 ± 1.5	14.1 ± 1.9	14.1 ± 1.9	14.2 ± 1.6	14.8 ± 2.1	15.2 ± 1.3
Dams with preimplantation losses/Examined dams	16/25	15/25	10/25	10/25	20/25	22/25	13/25	18/25
Preimplantation losses per dam (%)	8.8 ± 8.0	6.3 ± 6.5	5.6 ± 8.1	6.8 ± 12.9	12.5 ± 12.4	10.7 ± 7.0	7.9 ± 10.6	10.8 ± 9.2
Postimplantation losses during early pregnancy^b^	9	7	10	7	8	11	9	10
Postimplantation losses during middle pregnancy^c^	0	3	0	0	2	0	0	3
Postimplantation losses during late pregnancy^d^	0	0	0	0	2	0	2	0
Dams with postimplantation losses/Examined dams	7/25	8/25	9/25	7/25	9/25	9/25	8/25	10/25
Postimplantation losses per dam (%)	2.3 ± 4.0	2.8 ± 4.5	2.7 ± 3.9	1.9 ± 3.1	3.3 ± 6.2	3.1 ± 4.8	2.7 ± 4.3	3.4 ± 4.9
Dams with implantation losses/Examined dams	20/25	18/25	13/25	15/25	23/25	22/25	20/25	20/25
Total implantation losses per dam (%)	11.0 ± 7.8	8.9 ± 7.6	8.1 ± 9.3	8.7 ± 12.6	15.6 ± 12.2	13.5 ± 8.1	10.6 ± 9.7	13.7 ± 10.5
Live fetuses	368	349	349	345	340	345	360	368
Dams with live fetuses/Examined dams	25/25	25/25	25/25	25/25	25/25	25/25	25/25	25/25
Live fetuses per litter (%)	89.0 ± 7.8	91.1 ± 7.6	91.9 ± 9.3	91.3 ± 12.6	84.4 ± 12.2	86.5 ± 8.1	89.4 ± 9.7	86.3 ± 10.5
Live fetuses per litter	14.7 ± 1.2	14.0 ± 1.6	14.0 ± 1.5	13.8 ± 1.8	13.6 ± 2.0	13.8 ± 1.7	14.4 ± 1.8	14.7 ± 1.5
Male fetuses/Female fetuses	198/170	175/174	195/154	183/162	175/165	179/166	158/202	190/178
Male to fetuses per litter (%)	53.6 ± 12.8	49.9 ± 15.0	55.9 ± 14.0	53.0 ± 15.4	50.9 ± 12.4	52.1 ± 10.6	43.7 ± 10.1	51.7 ± 10.5*
Body weight of male fetus per litter (g)	3.91 ± 0.36	3.83 ± 0.25	3.85 ± 0.22	3.79 ± 0.22	3.87 ± 0.23	3.78 ± 0.23	3.92 ± 0.26	3.93 ± 0.20
Body weight of female fetus per litter (g)	3.70 ± 0.39	3.59 ± 0.25	3.64 ± 0.23	3.62 ± 0.22	3.70 ± 0.18	3.62 ± 0.23	3.71 ± 0.28	3.68 ± 0.22
Low-body-weight (<2.5 g) fetuses/Litters	1/1^e^	1/1	1/1	0/0	3/3	1/1	2/2	3/3
Placental weight of male fetus (mg)	475 ± 56	470 ± 49	455 ± 48	472 ± 61	450 ± 38	462 ± 53	463 ± 45	458 ± 49
Placental weight of female fetus (mg)	459 ± 74	439 ± 47	445 ± 55	460 ± 62	431 ± 43	428 ± 42	438 ± 40	439 ± 55

MF, magnetic field.

a Mean ± SD.

b Implantation sites on the endometrium or placental remnants smaller than 3 mm in diameter.

c Placental remnants larger than 3 mm in diameter or dead fetuses smaller than the placental diameter.

d Macerated fetuses or dead fetuses larger than the placental diameter.

e Total number of low-body-weight fetuses/total number of litters with at least one low-body-weight fetus.

* Significantly different from concurrent sham-exposed group (*p*<0.05).

The number of dams with postimplantation losses was comparable across the groups of all experiments. Postimplantation losses were predominant in the early phase of pregnancy, and the trend was the same across all experiments. Percentage of total (pre and post) implantation losses per dam did not indicate significant differences between the MF-exposed and the concurrent sham-exposed groups in any experiments.

### Litter Viability, Sex Ratio, Fetal Body Weight, and Placental Weight

Reproductive endpoints relating to the fetuses are also displayed in Table 2. The number of live fetuses per litter did not exhibit significant difference between MF-exposed and concurrent sham-exposed groups. A significant increase in male sex ratio was found only in rats from experiment 2 of 60 kHz MF exposure. In other experiments, no significant changes in the sex ratio were found. No significant changes were evident in body weight of male and female fetuses per litter, number of low-body-weight fetus, or placental weights of male and female fetuses across all experiments.

### Fetal Evaluation

Fetal abnormalities are explicitly described in Table 3. Nine scattered external malformations were found; none of these showed significant differences between the MF-exposed and the respective sham-exposed groups. Among the six recorded visceral malformations, the liver lobulation anomaly was the most frequent across all the experiments. None of these malformations showed significant group differences. Thirteen types of skeletal malformations were found in this study; however, only four fetuses were skeletally malformed. Three such fetuses were in the sham-exposed groups, two from experiment 1 of 60 kHz MF exposure and one from experiment 2 of 60 kHz MF exposure, whereas only one fetus from experiment 1 of the 20 kHz MF-exposed group displayed malformations. Overall, no group differences were evident.

**Table 3:** Fetal Abnormalities Observed in Litters Exposed to 20 or 60 kHz Magnetic Fields

	20 kHz, 0.2 mT(rms) Magnetic field				60 kHz, 0.1 mT(rms) Magnetic field			
		
	Experiment 1		Experiment 2		Experiment 1		Experiment 2	
				
Parameter	Sham-exposed	MF-exposed	Sham-exposed	MF-exposed	Sham-exposed	MF-exposed	Sham-exposed	MF-exposed
External finding								
Examined fetuses/Examined litters	368/25	349/25	349/25	345/25	340/25	345/25	360/25	368/25
External malformations in total	0/0^a^	2/2	1/1	0/0	2/2	1/1	0/0	0/0
Mandibular hypoplasia	0/0	1/1	0/0	0/0	0/0	0/0	0/0	0/0
Kinky tail	0/0	1/1	0/0	0/0	0/0	0/0	0/0	0/0
Ventral hernia	0/0	0/0	1/1	0/0	0/0	0/0	0/0	0/0
Cranioschisis	0/0	0/0	0/0	0/0	1/1	0/0	0/0	0/0
Micrognathia	0/0	0/0	0/0	0/0	1/1	0/0	0/0	0/0
Protruding tongue	0/0	0/0	0/0	0/0	1/1	0/0	0/0	0/0
Omphalocele	0/0	0/0	0/0	0/0	0/0	1/1	0/0	0/0
Rudimentary tail	0/0	0/0	0/0	0/0	1/1	0/0	0/0	0/0
Ectrodactyly	0/0	0/0	0/0	0/0	1/1	0/0	0/0	0/0
Visceral Finding								
Examined fetuses/Examined litters	178/25	168/25	170/25	165/25	164/25	167/25	175/25	178/25
Visceral malformations in total	1/1	4/4	4/3	1/1	5/5	3/3	3/3	6/5
Situs inversus	0/0	0/0	0/0	0/0	0/0	0/0	1/1	0/0
Persistent left umbilical artery	0/0	0/0	0/0	0/0	0/0	0/0	0/0	1/1
Diverticulum of small intestine	0/0	1/1	1/1	0/0	1/1	0/0	1/1	0/0
Lobulation anomaly of liver	0/0	3/3	3/2	0/0	3/3	3/3	1/1	4/3
Hydronephrosis	1/1	0/0	0/0	0/0	1/1	0/0	0/0	1/1
Diverticulum of large intestine	0/0	0/0	0/0	1/1	0/0	0/0	0/0	0/0
Skeletal Finding								
Examined fetuses/Examined litters	190/25	181/25	179/25	180/25	176/25	178/25	185/25	190/25
Skeletal malformations in total	0/0	1/1	0/0	0/0	2/2	0/0	1/1	0/0
Absence of parietal bone	0/0	0/0	0/0	0/0	1/1	0/0	0/0	0/0
Absence of interparietal bone	0/0	0/0	0/0	0/0	1/1	0/0	0/0	0/0
Shortening of mandibula	0/0	1/1	0/0	0/0	1/1	0/0	0/0	0/0
Absence of cervical vertebral arch	0/0	1/1	0/0	0/0	1/1	0/0	0/0	0/0
Absence of thoracic vertebral body	0/0	0/0	0/0	0/0	1/1	0/0	0/0	0/0
Fusion of cervical vertebral arches	0/0	1/1	0/0	0/0	0/0	0/0	1/1	0/0
Fusion of thoracic vertebral arches	0/0	1/1	0/0	0/0	1/1	0/0	0/0	0/0
Absence of lumbar vertebral body	0/0	1/1	0/0	0/0	1/1	0/0	0/0	0/0
Absence of lumbar vertebral arch	0/0	0/0	0/0	0/0	1/1	0/0	0/0	0/0
Agenesis of the sacro-coccygeal vertebrae	0/0	0/0	0/0	0/0	1/1	0/0	0/0	0/0
Absence of rib	0/0	0/0	0/0	0/0	1/1	0/0	0/0	0/0
Fusion of ribs	0/0	1/1	0/0	0/0	1/1	0/0	0/0	0/0
Absence of ulna	0/0	0/0	0/0	0/0	1/1	0/0	0/0	0/0
Skeletal variations in total	11/7	12/8	22/10	12/10	16/8	12/8	14/8	18/14
Splitting of cervical vertebral arch	1/1	1/1	0/0	0/0	2/2	0/0	1/1	2/2
Dumbbell shape of cervical vertebral arch	0/0	0/0	0/0	0/0	0/0	0/0	0/0	1/1
Shortening of cervical vertebral arch	0/0	1/1	0/0	0/0	0/0	0/0	2/2	2/2
Splitting of thoracic vertebral body	0/0	4/4	1/1	4/3	2/2	3/3	1/1	0/0
Dumbbell shape of thoracic vertebral body	0/0	5/5*	5/5	4/3	1/1	0/0	5/4	5/5
Deformity of thoracic vertebral body	0/0	1/1	0/0	0/0	0/0	0/0	0/0	0/0
Splitting of thoracic vertebral arch	0/0	1/1	0/0	0/0	0/0	0/0	0/0	1/1
Dumbbell shape of thoracic vertebral arch	0/0	0/0	0/0	0/0	0/0	0/0	0/0	1/1
20 thoracic and lumbar vertebrae	0/0	1/1	0/0	0/0	0/0	0/0	1/1	0/0
Cervical rib	0/0	0/0	0/0	0/0	0/0	0/0	0/0	1/1
Absence of 13th rib	0/0	0/0	0/0	1/1	0/0	0/0	0/0	0/0
Shortening of 13th rib	0/0	0/0	2/2	2/2	2/2	0/0	0/0	0/0
Extra 14th rib	0/0	0/0	0/0	0/0	1/1	0/0	1/1	1/1
Rudimentary 14th rib	10/6	5/5	14/5	3/3	11/5	9/5	5/4	9/7
Asymmetry or splitting of sternebrae	0/0	0/0	0/0	0/0	1/1	1/1	1/1	1/1
Fetuses with at least 3 variations	0/0	2/2	0/0	0/0	0/0	0/0	0/0	1/1
Ossification (Number of ossified vertebral bodies)								
Cervical vertebrae	0.4^b^ ± 0.6	0.5 ± 0.8	0.4 ± 0.6	0.5 ± 0.8	0.4 ± 0.4	0.3 ± 0.4	0.2 ± 0.3	0.2 ± 0.3
Sacral and coccygeal vertebrae	8.0 ± 0.6	7.8 ± 0.4	8.0 ± 0.4	8.0 ± 0.4	7.9 ± 0.4	7.6 ± 0.4**	7.6 ± 0.5	7.7 ± 0.3

MF, magnetic field.

a Fetuses with findings/Litters with findings.

b Mean per litter ± SD.

* Significantly different from concurrent sham-exposed group (*p*<0.05).

** Significantly different from concurrent sham-exposed group (*p*<0.01).

The incidence of fetal skeletal variations per litter increased in experiment 2 of 60 kHz MF exposure, but it was not significant (Table 3). Among the 15 types of recorded skeletal variations, splitting of the thoracic vertebral body, dumbbell shape of thoracic vertebral body, and a rudimentary 14th rib were relatively frequent in both MF-exposed and sham-exposed groups. A single type of variation (dumbbell shape of thoracic vertebral body) increased significantly only in the 20 kHz, MF-exposed group from experiment 1. No skeletal variations were found to have changed significantly in other experiments. Two types of skeletal ossifications were examined in this study (Table 3). Ossification of sacral and coccygeal vertebrae was found delayed in the 60 kHz, MF-exposed group from experiment 1. No other experiments showed such a significant difference.

### Paternal Toxicity

Unilateral kidney cysts were found in two mated males in experiment 1 of the 20 kHz MF exposure, one from the sham-exposed and another from the MF-exposed group. There was no evidence of gross findings in any other males.

## DISCUSSION

Because studies to evaluate the biological effects of IF MFs are sparse, a group of 25 pregnant rats were exposed to either 20 kHz, 0.2 mT(rms) or 60 kHz, 0.1 mT(rms) sinusoidal MFs during organogenesis. Experiments were duplicated, and teratological evaluations were performed in a blind fashion. No reproducible changes between sham-exposed and MF-exposed groups were found. The obtained data demonstrate that the IF MFs at either frequency were not teratogenic.

Increases in fetal loss and low-body-weight fetus by IF MF exposure were reported in two earlier studies. Frölen et al. (1993) found these changes in CBA/S mice exposed to 20 kHz, 15 µT(pp) MF from days 1 to 19 postconception. Although an additional study (Svedenstål and Johanson, 1995) failed to reproduce the increased fetal losses, it succeeded in revealing significant decreases in the body weight and length of living fetuses exposed to the MF during the preimplantation period (days 1.0–5.5 postconception). Their statistics, however, were based on a total number of fetal losses or implantations. Wiley et al. (1992) exposed CD-1 mice to 20 kHz, sawtooth MFs up to 0.2 mT(pp) from days 1 to 18 of pregnancy. Their primary unit of analysis was the litter and they found that MF exposure had no influence on numbers of implantations, fetal death or early loss, gross external, visceral, and skeletal malformations, or fetal weight.

We exposed pregnant rats to intense 20 and 60 kHz MF during organogenesis. As shown in Table 2, maximum postimplantation loss per litter was 3.3% and maximum litter number containing at least one low-body-weight (<2.5 g) fetus was as low as 3 per 25 among the sham-exposed groups. Results indicate that the IF MF affects neither implantation loss nor fetal body weight. However, because a relatively large portion of fetal loss occurs during the preimplantation period, the MF effect on early fetal loss should be investigated by exposure specific to the period.

Fetal malformations were closely studied by Huuskonen et al. They exposed Han:Wister rats to 20 kHz, 15 µT(pp) sawtooth MFs throughout the gestation days 0 to 20 (Huuskonen et al., 1993). Results showed no significant malformations in external, visceral, or skeletal examinations or in other reproductive indices affected by exposure. Although the numbers of variants (fetuses with at least three different variations as defined in the article) and incidence of skeletal anomalies (fetuses with minor skeletal malformations and/or variants) increased in the MF-exposed rats, the increases were not statistically significant when compared by litter.

Huuskonen et al. (1998b) also exposed CBA/Ca mice to the same IF MF from days 0 to 18 of pregnancy. The MF did not cause major or minor malformations in the fetuses, but increased variants and decreased ossification in the mice. The research group (Juutilainen et al., 1997; Huuskonen et al., 1998a) then used CBA/S mice (the strain that showed fetal loss in a study by Frölen et al., 1993) to reproduce minor skeletal abnormalities in CBA/Ca mice observed in their preceding study. The results were not in accord with their previous findings in the CBS/Ca mice. Suggested interpretation in the paper (Huuskonen et al., 1998a) was that the effects of MF exposure were strain-specific, although the plausible mechanism for the specificity remained unproven.

Examination of fetal abnormalities in this study shows no embryo/fetotoxicity or teratogenicity at either 20 or 60 kHz MFs. Incidence of external, visceral, and skeletal malformations were consistently low across the exposed and sham-exposed groups and no exposure-specific type of abnormality was manifest. The two exceptional fetuses bore most of the malformations seen in this study. The fetus in the 20 kHz MF-exposed group had low-body-weight with mandibular hypoplasia, and all six types of skeletal malformations in the group were found in this one fetus alone (Table 3). The other in the sham-exposed group in experiment 1 of the 60 kHz exposure exhibited similarly concentrated abnormalities. Because the incidence was so isolated, such abnormalities could be spontaneous.

Skeletal variations do not generally reflect significant teratogenicity. Stuchly et al. (1988) exposed SD female rats from 2 weeks before and throughout pregnancy for 7 hr/d to 17.86 kHz sawtooth MFs up to 0.66 mT(pp). They observed increased minor variations in skeletal development but interpreted them as commonly observed changes that appear in every teratological evaluation. Ryan et al. (2000) exposed SD pregnant rats from gestation days 6 to 19 to 60 Hz and/or its third harmonic 180 Hz MFs. They also found increased rib variants in the 60 Hz and superimposed 180 Hz MFs group, but the frequency of the variants was similar to that in a historical control from their laboratory.

In this study, a skeletal variation—dumbbell shape of thoracic vertebral body—showed a statistically significant increase in the 20 kHz MF experiment 1 (Table 3). The increase was not reproduced in other sets of experiments. Frequency of the incidence (five fetuses in 5/25 litters) was equivalent to the sham-exposed rats in other experiments. This suggests that this particular variation at the frequency up to 5/25 litters could be our historical incidence. The observed significant increase of the skeletal variation in the 20 kHz MF experiment 1 could be by chance because of the zero incidence in the corresponding sham-exposed group.

Huuskonen et al. (1998b) reported decreased ossification in proximal toes by IF MF exposure; whereas other studies did not indicate changes in fetal ossifications (Stuchly et al., 1988; Chiang et al., 1995; Huuskonen et al., 1998a). In this study, a statistically significant decrease in ossification of sacral and coccygeal vertebrae was found in the 60 kHz experiment 1. A fetus with decreased ossification is generally associated with developmental retardation. In this particular experiment, fetuses with such decreased ossification tended to have lower body weight compared to the group mean, but the mean body weights of fetuses per litter were comparable between all paired sham-exposed and MF-exposed groups. Because this lowered number of ossified vertebral bodies was not reproduced in the rest of the experiments and was not associated with changes in body weight of fetuses on a per litter basis, it does not suggest that MF interfered with fetal development.

Fetus sex ratio increased (fewer males to females) in the MF-exposed group in experiment 2 of the 60 kHz exposure. The ratio in the corresponding sham-exposed group was relatively low (43.7%) to that of sham-exposed groups in the rest of three experiments (50.9–55.9%). The reason for the low ratio is unknown. At least, it could be argued that the MF exposure did not affect the sex ratio of the fetus in the 60 kHz experiment 2 (51.7%), because it was located within the range of other sham-exposed groups and because the significant increase was not observed in other experiments in this study. Also no change in fetal sex ratio by IF MF exposure was reported in other studies (Huuskonen et al., 1993, 1998a,b; Lee et al., 2009).

Results from the hematological and blood chemistry examinations in pregnant rats showed no reproducible changes attributed to MF exposures. The observed alterations were sporadic and did not affect organogenesis of the fetuses exposed to IF MFs. Some IF MF studies have also reported isolated changes in dams' hematology or blood chemistry, but as in our study, the changes had no direct relevance to teratogenicity (Dawson et al., 1998; Huuskonen et al., 1998a; Kim et al., 2004).

In conclusion, exposure to 20 kHz, 0.2 mT(rms) and 60 kHz, 0.1 mT(rms) sinusoidal MFs during rat organogenesis displayed no reproducible embryo/fetotoxicity or teratogenicity under the present experimental conditions. No MF frequency-specific response was manifested in any of the endpoints. Therefore, the evidence does not support the hypothesis that IF MF exposure after implantation carries a significant risk for the developing mammalian fetus.
